# Heat Stress Increases Mammary Epithelial Cells and Reduces Viable Immune Cells in Milk of Dairy Cows

**DOI:** 10.3390/ani12202810

**Published:** 2022-10-17

**Authors:** Andrea J. Lengi, Jacob W. Stewart, Melissa Makris, Michelle L. Rhoads, Benjamin A. Corl

**Affiliations:** 1 Department of Dairy Science, College of Agriculture and Life Sciences, Virginia Tech, Blacksburg, VA 24061, USA; 2 Department of Animal and Poultry Sciences, College of Agriculture and Life Sciences, Virginia Tech, Blacksburg, VA 24061, USA; 3 Flow Cytometry Laboratory, Virginia-Maryland College of Veterinary Medicine, Virginia Tech, Blacksburg, VA 24061, USA

**Keywords:** heat stress, dairy cow, mammary epithelial cell

## Abstract

**Simple Summary:**

Heat stress has negative impacts on dairy production. This study examined the effect of heat stress on milk somatic cell populations in lactating dairy cows. The concentration and viability of mammary epithelial cells, T cells, monocyte/macrophage, and granulocytes in milk was compared in cows maintained under heat stressed versus thermoneutral conditions. Increases in the concentration of mammary epithelial cells and decreases in the concentration of live granulocytes and live total CD45^+^ cells in milk from heat stressed cows suggests a mechanism by which heat stress impacts milk production and immunocompetence of dairy cows.

**Abstract:**

Somatic cells normally found in milk are generally either immune cells such as lymphocytes, monocytes and granulocytes, or mammary epithelial cells. The number and composition of somatic cells in milk can be influenced by a variety of factors, including infection and temperature-humidity index. The objective of this study was to determine the specific effects of heat stress on the cellular composition of the somatic cell population in milk. We used flow cytometry to ascertain the concentration and viability of mammary epithelial cells, T cells, monocyte/macrophage, and granulocytes in milk from cows maintained under heat stressed conditions compared to thermoneutral conditions. We found a significant 10% increase in the natural log concentration of epithelial cells in the milk of heat stressed cows compared to thermoneutral cows (9.3 vs. 8.4 ln(cells/mL, *p* = 0.02)). We also found a 12% decrease in the log concentration of live CD45^+^ cells (*p* = 0.04), and a 17% decrease in the log concentration of live CD45^+^ granulocytes (*p* = 0.04). No changes were found in CD3^+^CD45^+^ cells or CD14^+^CD45^+^ cells, however, we noted an unusual population of CD14^+^CD45^−^ cells that showed significant increases of 10% (*p* = 0.03) and 12% (*p* = 0.01) in the log concentration of total and dead cells, respectively, under heat stressed conditions. These results suggest that heat stress influences the relative populations and viability of some somatic cells populations in milk. Increased losses of secretory epithelial cells into milk could have implications for milk production, and fewer viable immune cells could negatively impact the immunocompetence of dairy cows under heat stress.

## 1. Introduction

Somatic cells are found in milk from all mammals. Many factors influence the numbers and populations of somatic cells in cow’s milk, including breed, parity, lactation stage, health status, and season [1,2]. The populations of somatic cells found in milk are typically either immune cells of hematopoietic origin recruited from circulation, or mammary epithelial cells shed into milk. The immune cell component is predominantly T cells, myeloid cells such as monocytes and macrophage, and granulocytes such as neutrophils, eosinophils and mast cells [3]. Of the granulocytes, neutrophils are the most numerous in milk and serve as a line of defense against intramammary infection assisting in repair of damaged tissue [4]. T cells assist in both cell-mediated and humoral immunity, while macrophage and neutrophils phagocytose and destroy invading pathogens. In mastitic udders, the percentages of neutrophils in milk typically increase, with a corresponding decrease in the percentages of epithelial cells [5].

The mammary epithelial cells found in milk may result from shedding due to the pressures of repeated filling and emptying of the gland, or disruption of the mammary epithelium during milking, or may be part of a more regulated process of turnover of secretory tissue [6,7]. The percentage of mammary epithelial cells found in milk varies widely from study to study, and on the methods used. For example, using microscopy, Leitner et al., (2000) found that epithelial cells made up 45% of the somatic cells found in bovine milk from uninfected udders, whereas studies using antibody and magnetic bead purification methods have found much lower percentages [8].

Several studies show a strong correlation between increasing SCC in milk and increasing temperature-humidity index [9,10,11]. Changes in milk SCC due to heat stress could result from increased shedding of mammary epithelial cells into milk, as well as increased infiltration of immune cells into the mammary gland due to increased rates of infection with heat stress. However, increased cell numbers may not equate to increased function. Several in vitro studies have shown a direct negative effect of heat stress on bovine immune cell function. For example, peripheral blood mononuclear cells from Holstein and Brown Swiss cows showed reduced proliferation in culture when exposed to cycles of heat stress [12] and lymphocytes isolated from Holstein cows showed reduced proliferation when cultured under higher temperatures [13]. Further, phagocytic and burst activity of bovine polymorphonuclear cells decreased with increasing culture temperatures [14], and bovine lymphocytes exhibited a reduced response to mitogens in culture after heat shock [15].

Milk yield declines 25 to 40% during periods of heat stress due to decreased feed intake, decreased uptake of nutrients by the mammary gland, and increased apoptosis of mammary epithelial cells [11]. One possible contributing factor for decreased milk yield under heat stress conditions is an increase in the loss of epithelial cells into milk. Milk yield is governed by the number and activity of mammary epithelial cells [16]–a balancing act between proliferation and differentiation of new cells, and cell loss via apoptosis, or shedding of mammary epithelial cells into milk.

The objective of the current study was to examine the effects of heat stress in dairy cows on the numbers and viability of milk somatic cells. We employed flow cytometry, using a viability dye and antibodies specific for markers of cell types typically found in bovine milk, to assess the composition of milk somatic cell populations from cows housed in thermoneutral or heat stress conditions.

## 2. Materials and Methods

### 2.1. Animals and Experiment

This experiment utilized a subset of samples from a previous study [17] and complete experimental details can be found in that paper. All procedures involving animals were approved by the Virginia Tech Institutional Animal Care and Use Committee.

Twelve Holstein dairy cows at 55 ± 13 days in milk (DIM, mean ± SD) and of 3.0 ± 1.2 parity in three replicates from the six replicates of the full experiment were sampled. Only 12 cows were sampled due to unavailability of a fully functional flow cytometer during three of the six replicate experiments reported in [17]. Two cows of 12 were removed from one replicate experiment due to respiratory disease leaving incomplete data and their data were not included in analyses (n = 10).

A replicate experiment was 28 d, which included four experimental periods and two acclimation periods. Each experiment began with a 3-d period to acclimate cows to tie stalls and general husbandry procedures (d -3 to d -1). The first acclimation period began at the Virginia Tech Dairy Complex, after which cows were randomly paired and housed in one of two environmentally controlled rooms in Litton Reaves Hall. Cows in both rooms moved through sequential treatment periods simultaneously. Acclimation was immediately followed by the first two experimental periods, consisting of 1) thermal neutral (TN; d 1–5) conditions, and 2) thermal neutral and hyperinsulinemic-hypoglycemic clamp (d 6–10), after which the room temperature was increased and cows were allowed five days of acclimation to the new thermal conditions. The third and fourth experimental periods consisted of 3) heat stress (HS; d 16–20), and 4) heat stress and euglycemic clamp (d 21–25). Rectal temperatures (°C) and respiration rates (breaths/min) were measured once daily (12:30 h) during TN periods and three times daily (00:30, 09:00, 12:30 h) during HS periods. Temperature Humidity Index (THI) was calculated using dry bulb temperature (Tdb, °F) and relative humidity (RH) using the formula (Tdb-(0.55-(0.55*RH/100)*(Tdb-58). A THI of 68 or less was considered to be thermoneutral [18]. In the current study, the mean daily THI was 65.1 ± 0.2 during the TN periods. During the HS period, the room reached a maximum THI of 76.8 ± 0.5 in the afternoon and cooled to a minimum of 70.3 ± 0.4 overnight, for a mean daily THI of 72.5 ± 0.2 during the HS periods. For the current study, no samples were collected from the two clamp periods, which were part of a separate experiment. The clamp periods had no impact on somatic cell counts. Feed intake, milk yield, and milk component data as well as additional details on temperature programs can be found in Stewart et al., 2022 [17].

### 2.2. Milk Sampling and Processing

Cows were milked at 01:00 and 13:00 using a portable milking system and milk weights were recorded. Milk was sampled at the 01:00 milking of the fifth day of TN (d 5) or HS (d 20) for milk somatic cell characterization. On the days of milk somatic cell isolation, milk samples were preserved with 2-bromo-2-nitropopane-1,3-diol (Bronopol, D&F Inc. Dublin, CA, USA) and sent to Lancaster DHIA (Manheim, PA, USA) for analysis of SCC, fat, protein and other solids.

Milk was processed for cell population analysis as described previously [19]. Briefly, approximately 3.8 L of milk from each cow, containing a final concentration of 0.5 mM EDTA, was centrifuged at 600× *g* for 10 min. The resulting pellet was washed once with DPBS containing a final concentration of 0.5 mM EDTA, and then resuspended in Red Blood Cell Lysis Buffer (154 mM NH_4_Cl, 10 mM KHCO_3_, 0.1 mM EDTA) for 15 min at room temperature. To remove cell clumps and non-cellular debris, cells were filtered sequentially through 100 and 40 micron cell strainers, followed by centrifugation with Debris Removal Solution (Miltenyi Biotec, Auburn, CA, USA) according to manufacturer’s instructions. Cells were then counted using a hemocytometer, and 2 × 10^6^ cells were added to each tube for staining.

Cells were incubated with primary antibodies shown in Table 1. Butyrophilin 1A1 (BTN), was used as a specific marker for mammary epithelial cells [19]. CD45 was used as a general marker for the immune cell component of milk somatic cells. CD3, part of the T cell receptor complex, was used as a marker for T cells. CD14 was used as a marker for macrophage and monocytes. An antibody specific for bovine granulocytes was used to detect neutrophils and other granulocytic cells.

For each cow at each time point, cells were double labeled as follows: (1) BTN (APC) + CD45 (PE); (2) CD45 (PE) + CD3 (AF488); (3) CD45 (PE) + Granulocyte (AF488); (4) CD45 (PE) + CD14 (AF488). All samples were also stained with Hoechst33342 to label nuclei, and propidium iodide to distinguish live and dead cells. Cells were incubated in primary antibody at the indicated concentrations (Table 1) for 40 to 60 min in 100 µL of Cell Staining Buffer (Invitrogen) at room temperature and protected from light. Cells were washed with DPBS, collected by centrifugation at 600× *g* for 10 min, and then incubated in secondary antibody at the indicated dilutions (Table 1) for 40 to 60 min in 100 µL of Cell Staining Buffer at room temperature and protected from light. Cells were washed as before, and then resuspended in Hoechst 33342 and Propidium Iodide (PI; Table 1) for 40 to 60 min in 100 µL of DBPS at room temperature and protected from light. After a final wash, cells were resuspended in 100 µL of Cell Staining Buffer and analyzed by flow cytometry.

### 2.3. Flow Cytometry Analysis

Using the BD FACSAria Fusion Cell Sorter, an initial gate was drawn based on FSC-A and SSC-A, after which doublet exclusion was performed to eliminate aggregates using FSC-H/W and SSC-H/W parameters. Nucleated cells were selected by gating on Hoechst positive events. To assess total BTN^+^ and CD45^+^ cells, double staining was performed with an antibody specific for BTN1A1 directly conjugated to allophycocyanin (APC), and an antibody specific for CD45, used with a secondary antibody conjugated to phycoerythrin (PE). To determine the subsets of CD45^+^ cells, double staining was done with an antibody specific for CD45, used with a secondary antibody conjugated to PE, and antibodies specific for either CD3, CD14, or bovine granulocytes, all used with a secondary antibody conjugated to AlexaFluor488 (AF488). The viability stain PI was used in all experiments to determine live and dead percentages for each cell population.

### 2.4. Statistical Analyses

Cell populations were expressed as live and dead (PI^−^ vs. PI^+^) percentages of total cells. Concentrations of individual cell populations in milk somatic cells were calculated by multiplying somatic cell count (expressed in thousands of cells per mL) by cell population percentages.

Data were analyzed using the Mixed procedure of SAS (v. 9.4, Cary, NC, USA). Somatic cell count and somatic cell population data were natural log transformed to achieve equal variance and normality. All data were analyzed using a model that included treatment, replicate, and treatment by replicate interaction. Cow was included as a random term. Studentized residuals were reviewed, and any data point greater than 3.5 was removed as an outlier from the data set. This resulted in the removal of one data point for PI^+^Granulocyte^+^CD45^+^ cells from one cow during the heat stress treatment. Least square means and SEM of natural log transformed cell population data are presented. Significance was declared at *p* < 0.05 and tendencies were highlighted for differences at *p* < 0.10.

## 3. Results

Cow respiration rates and rectal temperatures are reported in a previous paper, and were both significantly increased during HS periods compared to TN periods [17]. Milk yield, components, and somatic cell count in cows maintained under thermoneutral and heat stress conditions are presented Table 2. Milk yield was significantly decreased by heat stress compared to thermoneutral conditions (*p* = 0.001), while somatic cell count was not changed by treatment. While we saw a decrease in milk fat (*p* = 0.02) and protein yield (*p* = 0.003) consistent with the decrease in milk yield, there was no change in milk fat or protein percentage between treatments. There was, however, a significant decrease in the percent (*p* = 0.02) and yield (*p* = 0.001) of other solids in the milk from heat stressed cows.

**Figure 1:**
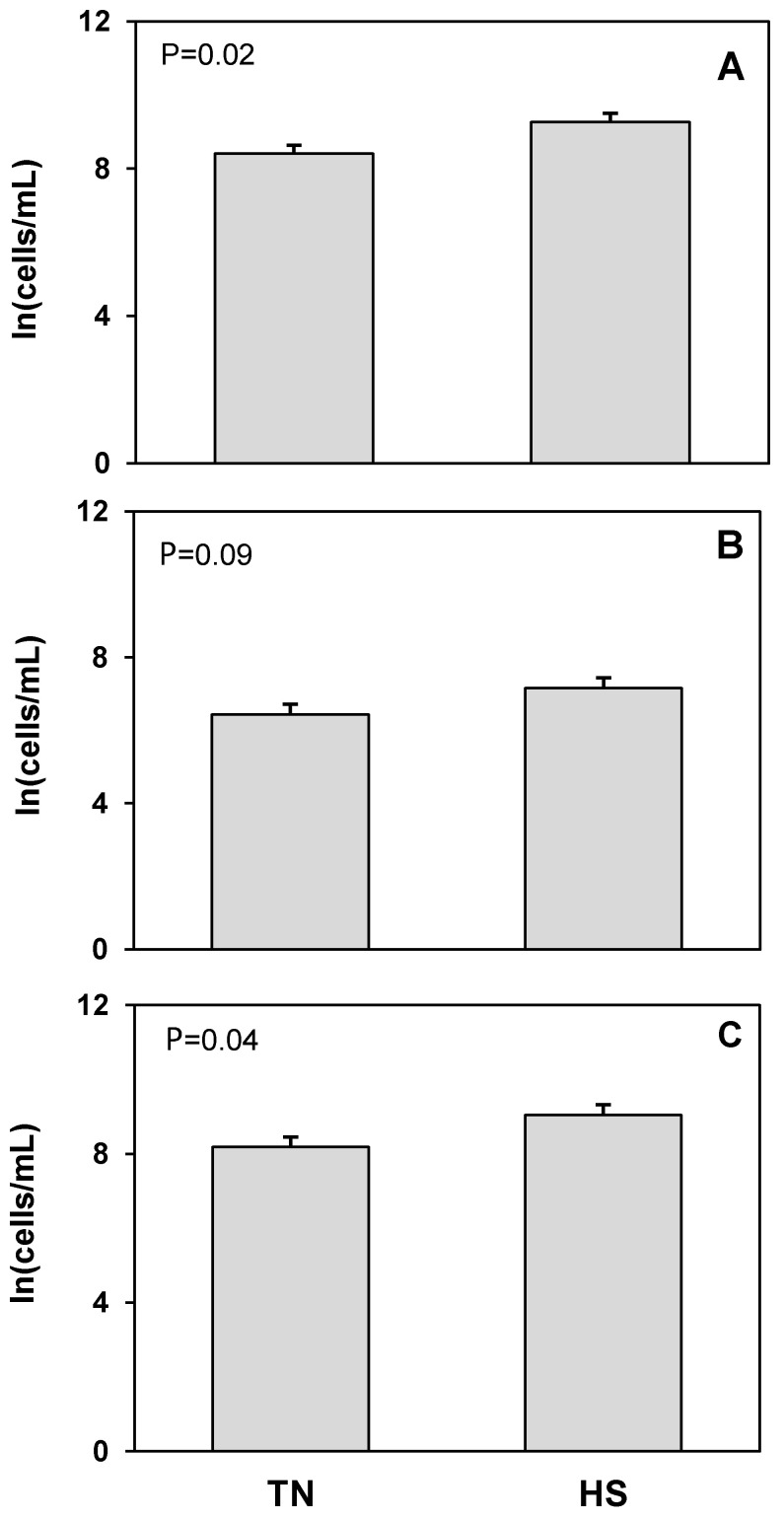
Heat stress (HS) impacts on mammary epithelial cells (butyrophilin positive; BTN^+^CD45^−^) cell concentration in milk compared to thermoneutral (TN) conditions. (**A**) Total cells, (**B**) live cells (propidium iodide negative; PI^−^), and (**C**) dead cells (propidium iodide positive; PI^+^). Cell population data were natural log transformed for statistical analysis. Values presented are least square means of natural log transformed data with error bars presenting the SEM (n = 10).

Butyrophilin was used as a flow cytometric marker for detecting mammary epithelial cells in milk. There was a 10% increase in the log transformed concentration of BTN^+^ cells, corresponding to an 82% increase in the untransformed concentration of BTN^+^ cells, in milk from heat stressed cows compared to milk from the same cows maintained under thermoneutral conditions (*p* = 0.02; Figure 1). There was a tendency for an increase in live (*p* = 0.09) and a significant increase in the log concentration of dead BTN^+^ stained cells in milk from heat stressed cows (*p* = 0.04; Figure 1) that reflected a 78% increase in the untransformed concentration of dead BTN^+^ cells in milk.

**Figure 2:**
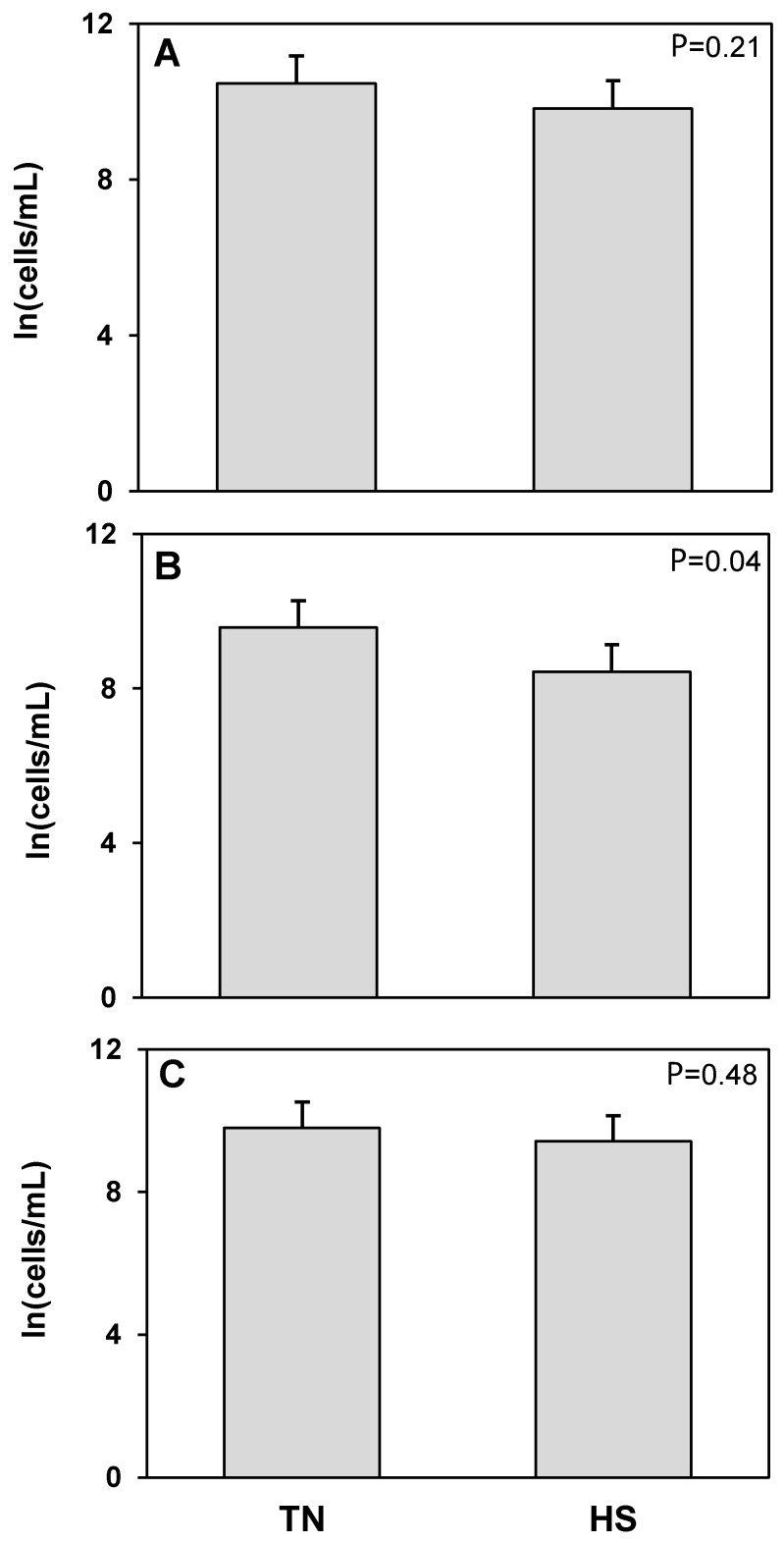
Heat stress (HS) impacts on hematopoietic (CD45^+^BTN^−^) cell concentration in milk compared to thermoneutral (TN) conditions. (**A**) Total cells, (**B**) live cells (propidium iodide negative; PI^−^), and (**C**) dead cells (propidium iodide positive; PI^+^). Cell population data were natural log transformed for statistical analysis. Values presented are least square means of natural log transformed data with error bars presenting the SEM (n = 10).

Using CD45 as a flow cytometric marker for immune cells in milk, we found no treatment differences in total number of CD45^+^ cells or dead CD45^+^ cells in milk (Figure 2). There was, however, a 12% decrease in the log of the concentration of live CD45^+^ cells in the milk of cows undergoing heat stress, compared to milk from the same cows at thermoneutral conditions (*p* = 0.04) reflecting a 71% decrease in the untransformed concentration of live CD45^+^ cells. To further elucidate what cell populations may account for this difference, we examined the different types of hematopoietic cells typically found in milk.

**Figure 3:**
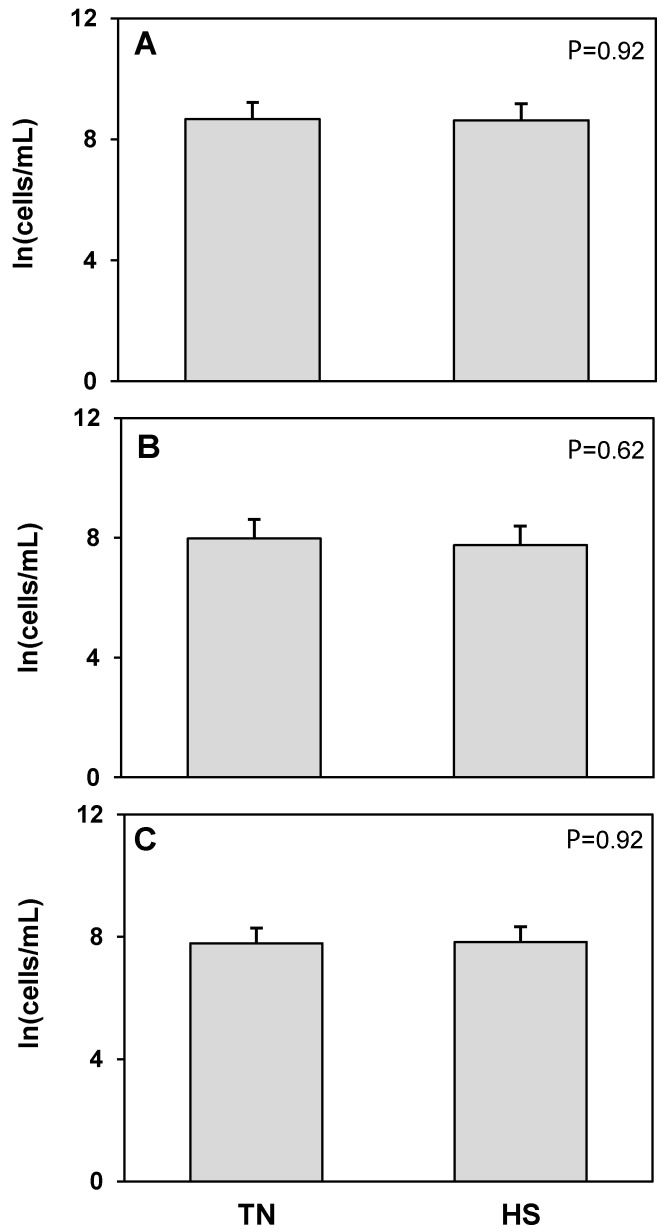
Heat stress (HS) impacts on lymphocytes (CD3^+^CD45^+^) cell concentration in milk compared to thermoneutral (TN) conditions. (**A**) Total cells, (**B**) live cells (propidium iodide negative; PI^−^), and (**C**) dead cells (propidium iodide positive; PI^+^). Cell population data were natural log transformed for statistical analysis. Values presented are least square means of natural log transformed data with error bars presenting the SEM (n = 10).

**Figure 4:**
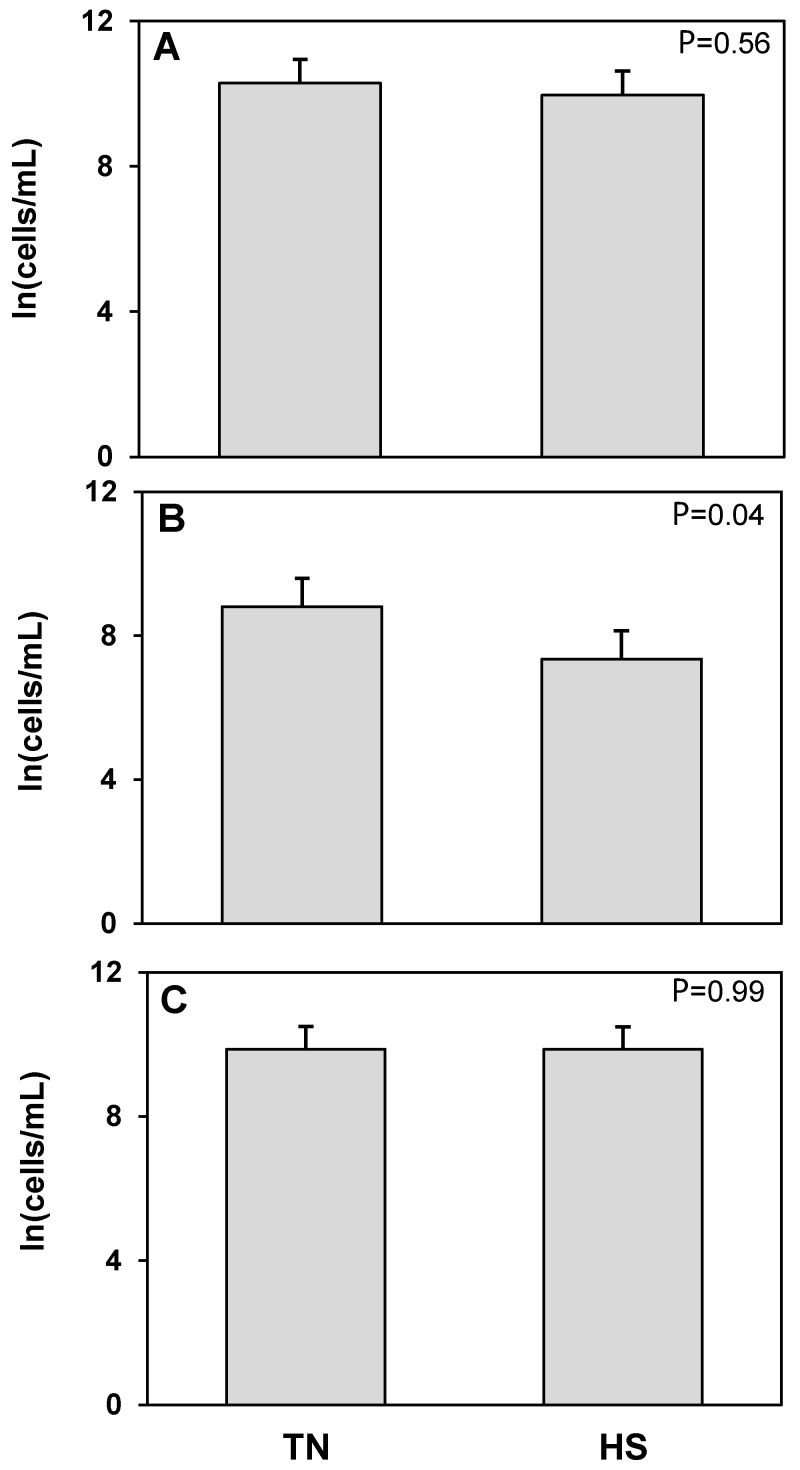
Heat stress (HS) impacts on granulocyte (Granulocyte^+^CD45^+^) cell concentration in milk compared to thermoneutral (TN) conditions. (**A**) Total cells, (**B**) live cells (propidium iodide negative; PI^−^), and (**C**) dead cells (propidium iodide positive; PI^+^). Cell population data were natural log transformed for statistical analysis. Values presented are least square means of natural log transformed data with error bars presenting the SEM (n = 10).

**Figure 5:**
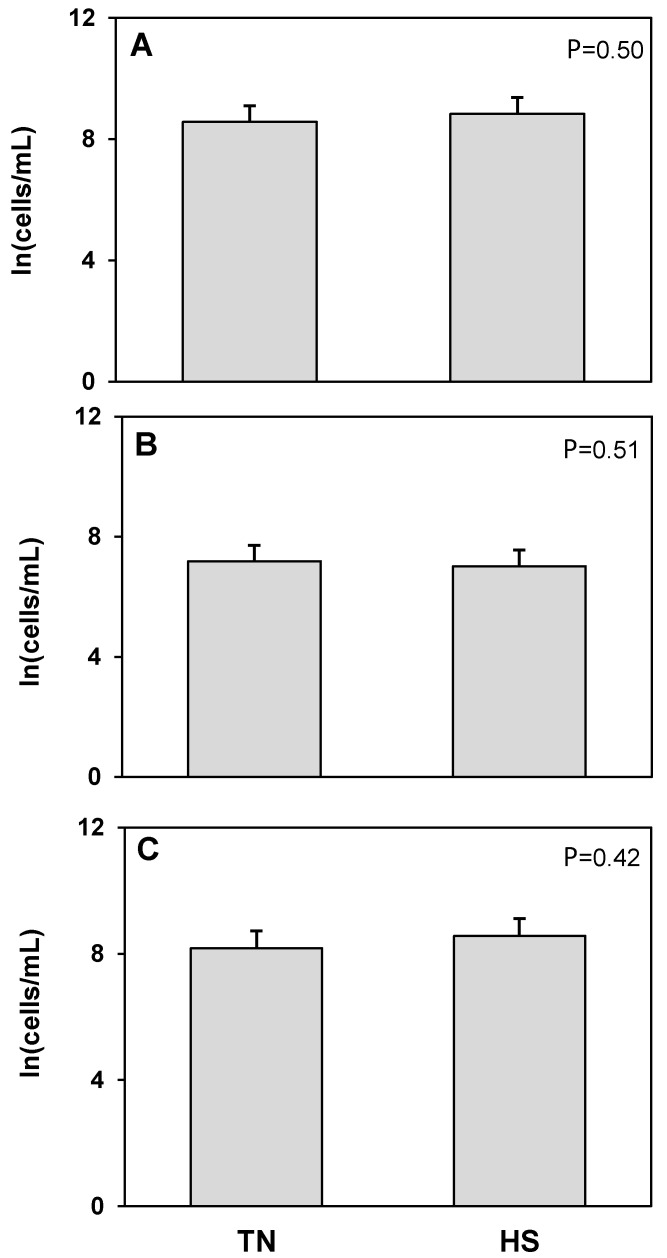
Heat stress (HS) impacts on monocyte/macrophage (CD14^+^CD45^+^) cell concentration in milk compared to thermoneutral (TN) conditions. (**A**) Total cells, (**B**) live cells (propidium iodide negative; PI^−^), and (**C**) dead cells (propidium iodide positive; PI^+^). Cell population data were natural log transformed for statistical analysis. Values presented are least square means of natural log transformed data with error bars presenting the SEM (n = 10).

**Figure 6:**
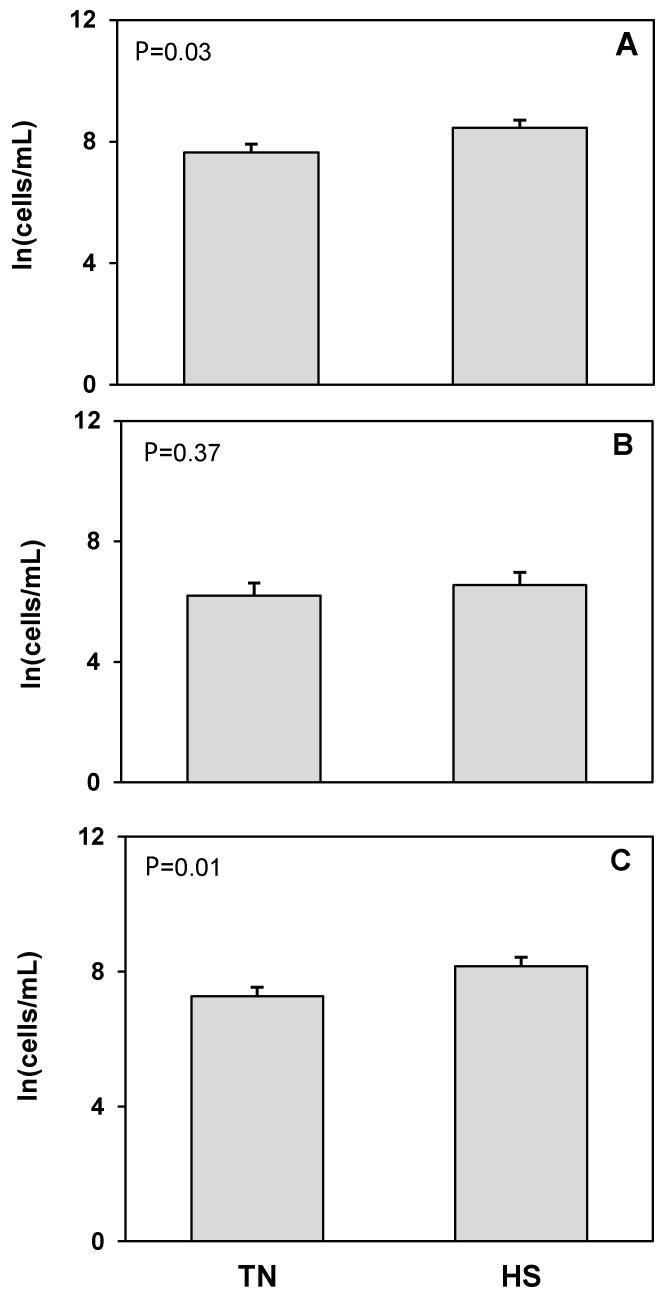
Heat stress (HS) impacts on CD14 positive, but CD45 negative (CD14^+^CD45^−^) cell concentration in milk compared to thermoneutral (TN) conditions. (**A**) Total cells, (**B**) live cells (propidium iodide negative; PI^−^), and (**C**) dead cells (propidium iodide positive; PI^+^). Cell population data were natural log transformed for statistical analysis. Values presented are least square means of natural log transformed data with error bars presenting the SEM (n = 10).

The T cell receptor protein CD3 was used as a marker to detect T cells. No treatment differences were found in the concentration of total, live, or dead CD3^+^CD45^+^ cells (Figure 3). An antibody raised against bovine granulocytes was used to detect neutrophils and other granulocytic cell populations such as mast cells and eosinophils. There were no treatment differences in total or dead granulocyte populations (Figure 4). The log concentration of live granulocytes decreased 17%, corresponding to a 77% decrease in the untransformed concentration, in milk from heat stressed cows compared to cows maintained under thermoneutral conditions (*p* = 0.04). CD14 was used as a marker for cells of macrophage and monocyte lineage. No treatment differences were found in total, live, or dead CD14^+^CD45^+^ cell populations (Figure 5). However, we found that in cells isolated from milk, there was a consistent, distinct population of CD14^+^CD45^−^ cells which has not been described in blood or other tissues. There was a significant 25% increase in the log concentration of total CD14^+^CD45^−^ cells, which was a 60% increase in the untransformed cell concentration, in response to heat stress (*p* = 0.03), which was driven by a 12% increase in the log concentration of dead CD14^+^CD45^−^ cells (*p* = 0.01) or 71% increase for the untransformed concentration. There was no treatment effect of heat stress on live CD14^+^CD45^−^ cell concentrations (Figure 6).

## 4. Discussion

We found milk yield to be significantly decreased by heat stress compared to thermoneutral conditions, in agreement with other studies examining the effects of heat stress in dairy cows and other ruminants [20,21]. We did not see a change in somatic cell count due to heat stress, in contrast to some studies that found increases during months with high temperature-humidity indices compared to winter months [9,10]. However, our results are consistent with other studies that found that increases in SCC during heat stress are not seen when cows are housed in environmentally controlled chambers as opposed to field conditions [22,23]. This could indicate that the increase in SCC seen during warmer months in some studies may not be entirely due to temperature, rather that conditions during warmer months are more conducive to pathogen proliferation, and cows may simply have more exposure to contamination during warmer months [24]. We found decreases in milk fat and protein yield, but not percentage, in the milk from heat stressed cows. Other researchers have seen varying effects on protein and fat content during heat stress, with some studies finding decreases in milk fat and protein content [25] and decreases in milk fat percentage in heat stressed cows [24], and others finding no change in fat percentage [26], or fat and protein percentage [27].

We have previously validated butyrophilin 1A1 as a marker for detecting mammary epithelial cells in milk [19]. Butyrophilin, one of a family of at least 10 genes in the Ig superfamily, is a transmembrane protein highly expressed in lactating mammary epithelium, and is critical for the packaging and secretion of milk fat globules into milk [28]. We found a significant increase in the log transformed concentration of total and dead BTN^+^ cells in milk from heat stressed cows compared to milk from the same cows maintained under thermoneutral conditions, and a tendency for an increase in log concentration of live BTN^+^ cells. Previous studies have shown that milk yield is governed by the number of mammary epithelial cells as well as the level of secretory activity per cell [29]. These studies concluded that the gradual decrease in milk yield after peak lactation can be accounted for almost entirely by cell loss via apoptosis, and that the effect of epithelial cell shedding into milk is negligible relative to rates of daily apoptosis assuming a constant epithelial cell content in milk somatic cells of 20% [16,29]. Other researchers, however, have estimated that 390 × 10^6^ epithelial cells are shed into milk every day, accounting for 1.6% of the total mammary epithelial cells in the udder [6]. Using concentrations of shed epithelial cells in combination with milk volume, we calculated that shedding of epithelial cells in milk in our study was 120 × 10^6^ cells per milking under thermoneutral conditions, and 221 × 10^6^ cells per milking under heat stress (*p* = 0.06). Another study found that feed restriction resulted in both decreased milk yield and increased rate of MEC shedding (59.9 vs. 98.7 × 10^6^ cells/d) [30]. Our findings in combination with those of others indicate that mammary epithelial cell content in milk somatic cells may not be a constant proportion of milk somatic cells and could be influenced by the environment. It is not clear whether the effect of heat stress on mammary epithelial cell shedding is a direct effect of heat stress on the mammary epithelium, or a secondary effect of decreased feed intake resulting in decreased milk yield and less need for, or less ability to support, as many secretory cells.

CD45, a transmembrane glycoprotein found on the surface of all nucleated hematopoietic cells and their precursors [31,32], was used as a marker for all hematopoietic cells in milk. While there were treatment differences in total number of CD45^+^ cells or dead CD45^+^ cells, there was a significant decrease in the log of the concentration of live CD45^+^ cells in the milk of heat stressed cows. As no differences were found in the concentration of total, live, or dead CD3^+^CD45^+^ cells, it does not appear that changes in T cell populations can account for this decrease. In addition to assisting in both cell-mediated and humoral immunity, T cells found in milk may directly transfer short-term passive cellular immunity to offspring, as well as directing the development of T cell repertoire in offspring. Studies in mice showed that of all the immune cell populations found in milk, only maternal T cells could be found in offspring tissues [33].

We found no treatment differences in total or dead granulocyte populations, while the log concentration of live granulocytes was significantly decreased in milk from heat stressed cows. Neutrophils are the most numerous type of granulocyte found in milk somatic cells, and are recruited to sites of infection, including the mammary gland, by chemoattractants such as interleukin-8 (IL-8). IL-8 plays a major role in recruiting granulocytes to the mammary gland during mastitis, and chemotactic activity can be blocked by neutralizing antibodies against IL-8 [34]. In response to chemoattractants generated in response to mammary infection, granulocytes migrate from the blood through the endothelium matrix, basement membrane, and mammary epithelium and into the milk, where they phagocytose and destroy pathogens. The act of migration is energy intensive, and once in the milk, granulocytes phagocytose fat and casein, which further reduce their potential phagocytic activity. Therefore, it is hypothesized that large numbers of granulocytes are needed in the mammary gland to provide efficient protection against intramammary infection [3]. Reductions in the concentration of viable granulocytes in the mammary gland could indicate that the ability of the mammary gland to respond to infection is compromised during heat stress. Simple evaluation of granulocyte concentration within milk somatic cells would not reveal this without the addition of a viability stain. Additional examination of the responsiveness of this cell population in milk to challenge is warranted to assess the full implications of the reduction of viable granulocytes in milk on the ability of the gland to defend against pathogens.

CD14, a protein expressed on the surface of myeloid cells (macrophage and monocytes) that functions to detect and bind bacterial lipopolysaccharide (LPS) [35], was used as a marker for cells of macrophage and monocyte lineage. Monocytes and macrophage are mononuclear phagocytic leukocytes which are recruited to sites of infection by chemokines such as Chemokine (C-C Motif) ligand 5 (CCL5) and Monocyte Chemoattractant Protein-1 (MCP-1). They have the capacity to secrete a wide variety of immunomodulatory molecules in addition to engulfing invading pathogens [36]. When exposed to LPS, endothelial cells produce cytokines and chemokines such as IL-8 and MCP-1, which serve to recruit neutrophils and monocytes/macrophage, respectively, to sites of infection [37]. CD14 acts as a co-receptor with Toll-like receptor (TLR) 4 and lipopolysaccharide binding protein (LBP) to bind LPS and activate cells to aid in bacterial clearance. While no treatment differences were found in CD14^+^CD45^+^ cell populations, we found a significant increase in the log concentration of a novel population of CD14^+^CD45^−^ cells in response to heat stress, which was driven by an increase in the log concentration of dead CD14^+^CD45^−^ cells.

In addition to being a membrane bound protein on the cell surface of monocytes and macrophage, CD14 also exists as a 48 kDa soluble form found in bodily fluids [38,39]. It binds to LPS of Gram-negative bacteria and can enhance signaling through Toll-like receptors in response to bacterial infections [40]. The soluble form of CD14 can be found circulating in blood, but it is found at a much higher concentration in milk, and is thought to function to bind and sequester LPS from CD14 and TLR-expressing immune cells, thereby allowing for clearance of LPS from the body while reducing the risk of septic shock [41]. The high concentrations of soluble CD14 in milk are thought to result in absorption of CD14 intact along the gut of nursing offspring, aiding in passive immunity transferred from the mother, and immune system stimulation and surveillance in the infant [42,43,44]. Soluble CD14 bound to LPS and LBP can then bind to endothelial and epithelial cells to activate cytokine secretion [45] thereby allowing nonmyeloid cells a way to respond to bacterial infections. It is possible that the CD14^+^CD45^−^ population of cells seen in milk may be soluble CD14 bound to a nonmyeloid cell such as mammary epithelial cells.

Further, several studies have shown that CD14 can be expressed in mammary epithelial cells at various stages of development. Two recent single cell RNA seq studies found CD14 mRNA expression in luminal progenitor cells and maturing epithelial cells [46,47], while another study found that CD14 expression is upregulated on the apical surface of mammary epithelial cells themselves, from 24 h after the start of involution and persisting through the first 4 days [48]. It has been hypothesized that this expression of CD14 on mammary epithelial cells indicates the development of a phagocytic phenotype in these cells [48,49], facilitating clearance of shed cells during involution [50]. Future work will examine these possible sources of the CD14^+^CD45^−^ population of cells found in bovine milk.

## 5. Conclusions

Heat stress in a controlled, experimental environment reduced milk yield in early lactation dairy cows without affecting somatic cell count. The concentration of mammary epithelial cells in somatic cells detected through expression of BTN by flow cytometry increased with heat stress, and trends were detected for increased concentration of both live and dead BTN^+^ cells. The increased concentration of BTN^+^ cells in milk somatic cells could indicate a direct response of mammary epithelial cells to heat stress or a regulatory mechanism related to reduced milk yield with heat stress. Additionally, the concentration of viable CD45^+^ immune cells in milk somatic cells was reduced with reduction in viable granulocytes accounting for this. The overall concentrations of CD3^+^CD45^+^ and CD14^+^CD45^+^ in milk somatic cells and their viability were unaffected by heat stress, but a population was of CD14^+^CD45^−^ cells were detected in small concentrations in milk somatic cells and the concentration of total and dead cells increased in milk somatic cells with heat stress. Overall, these results show that heat stress influences the relative populations and viability of some somatic cells populations in milk. Increased losses of epithelial cells into milk suggests a mechanism by which heat stress in lactating cows could negatively affect milk production, while fewer viable immune cells could impact immunocompetence of dairy cows under heat stress, leading to increased susceptibility to mammary infection.

## Figures and Tables

**Table 1:** Antibodies used to label milk somatic cell populations for flow cytometry analyses.

Antibody Target	Catalog Number	Clone	Concentration
Primary Antibodies			
BTN1A1	MAB8467APC ^a^	2151C	7.0 ng/µL
CD45	WS0544B-100 ^b^	CACTB51A	3.1 ng/µL
CD3	WS0561B-100 ^b^	MM1A	6.25 ng/µL
Granulocyte	WS0609B-100 ^b^	MM20A	1.25 ng/µL
CD14	WS0564B-100 ^b^	CAM36A	1.25 ng/µL
Secondary Antibodies			
Rat anti-mouse IgG2a-PE	1155-09 ^c^	SB84a	1.0 ng/µL
Goat anti-mouse IgG1-AF488	1070-30 ^c^	Polyclonal	1.25 ng/µL
Stains			
Hoechst33342	H3570 ^d^		1:1000
Propidium Iodide	556463 ^e^		1:10

^a^ Novus Biologicals. ^b^ Kingfisher Biotech, Inc. ^c^ Southern Biotech Associates. ^d^ Invitrogen (ThermoFisher Scientific). ^e^ BD.

**Table 2:** Milk yield and components of lactating dairy cows (n = 10) in thermoneutral (TN) or heat stress (HS) conditions for 5 days ^a^.

	TN	HS	SEM	*p*
Milk yield (kg per milking)	28.2	21.4	1.29	0.001
Milk fat				
%	4.23	3.88	0.33	0.48
g per milking	1163	836	89	0.02
Milk protein				
%	2.61	2.52	0.06	0.28
g per milking	737	536	32	0.003
Milk other solids				
%	5.87	5.73	0.04	0.02
g per milking	1656	1227	78	0.001
Somatic Cells ^b^				
Ln(cells per mL)	4.17	4.24	0.54	0.72

^a.^Data are reported as least square means. ^b.^Values are least square means of natural log transformed data.

## Data Availability

The data that support this study will be shared upon reasonable request to the corresponding author.
